# Contemporary diagnosis and treatment of valvular heart disease in Korea: a nationwide hospital-based registry study

**DOI:** 10.1186/s44348-024-00036-z

**Published:** 2024-11-22

**Authors:** Hyung Yoon Kim, Hee Jeong Lee, In-Cheol Kim, Jung-Woo Son, Jun-Bean Park, Sahmin Lee, Eun Kyoung Kim, Seong-Mi Park, Woo-Baek Chung, Jung Sun Cho, Jin-Sun Park, Jeong-Sook Seo, Sun Hwa Lee, Byung Joo Sun, Chi Young Shim, Hyungseop Kim, Kye Hun Kim, Duk-Hyun Kang, Jong-Won Ha, on behalf of the Korean Valve Survey Registry Investigators, Wook-Jin Chung, Chan Seok Park, Hyo-Suk Ahn, Eun Joo Cho, Dong Ryeol Ryu, Dong Heon Yang, Jeong Rang Park, Woo-Shik Kim, Il Suk Sohn, Jin Oh Na, Hwang Sun Ho, Choi Ji-Yong, Tae-Ho Park, Yong Hyun Park, Jung Hyun Choi, Hack-Lyoung Kim, Hye Sun Seo, Eui-Young Choi, Jang-Won Son, Shin-Jae Kim, Sang Jae Rhee, In-Jeong Cho, Young Sup Byun, Sung-Hee Shin, Se-Jung Yoon, Jong Wook Beom, Ju-Hee Lee, Dae-Hwan Bae, Sung-Ai Kim, Dae Gyun Park, Min-Kyung Kang, Kyung-Soon Hong, Ran Heo

**Affiliations:** 1 Department of Cardiovascular Medicine, Chonnam National University Hospital, Chonnam National University Medical School, Gwangju, Republic of Korea; 2 Division of Cardiology, Department of Internal Medicine, Cardiovascular Center, Keimyung University Dongsan Hospital, Keimyung University School of Medicine, Daegu, Republic of Korea; 3 Division of Cardiology, Department of Internal Medicine, Yonsei University Wonju College of Medicine, Wonju, Republic of Korea; 4 Division of Cardiology, Department of Internal Medicine, Seoul National University Hospital, Seoul, Republic of Korea; 5 Division of Cardiology, Heart Institute, Asan Medical Center, University of Ulsan College of Medicine, Seoul, Republic of Korea; 6 Division of Cardiology, Department of Medicine, Samsung Medical Center, Sungkyunkwan University School of Medicine, Seoul, Republic of Korea; 7 Division of Cardiology, Department of Internal Medicine, Korea University Anam Hospital, Korea University College of Medicine, Seoul, Republic of Korea; 8 Division of Cardiology, Department of Internal Medicine, Seoul St. Mary’s Hospital, College of Medicine, The Catholic University of Korea, Seoul, Republic of Korea; 9 Division of Cardiology, Department of Internal Medicine, Daejeon St. Mary’s Hospital, College of Medicine, The Catholic University of Korea, Daejeon, Republic of Korea; 10 Department of Cardiology, Ajou University School of Medicine, Suwon, Republic of Korea; 11 Division of Cardiology, Department of Internal Medicine, Inje University Busan Paik Hospital, Busan, Republic of Korea; 12 Division of Cardiology, Department of Internal Medicine, Chonbuk National University Medical School, Jeonju, Republic of Korea; 13 Division of Cardiology, Severance Cardiovascular Hospital, Yonsei University College of Medicine, Seoul, Republic of Korea

**Keywords:** Heart valve diseases, Diagnosis, Treatment, Outcome, Korea

## Abstract

**Background:**

This study was designed to determine the current status of diagnosis and treatment of valvular heart disease (VHD) in Korea.

**Methods:**

A nationwide registry study was conducted in 45 hospitals in Korea involving adult patients with at least moderate VHD as determined by echocardiography carried out between September and October of 2019. Of a total of 4,094 patients with at least moderate VHD, 1,482 had severe VHD (age, 71.3 ± 13.5 years; 49.1% male). Echocardiographic data used for the diagnosis of each case of VHD were analyzed. Experts from each center determined the diagnosis and treatment strategy for VHD based on current guidelines and institutional policy. The clinical outcome was in-hospital mortality.

**Results:**

Each valve underwent surgical or transcatheter intervention in 19.3% cases of severe mitral stenosis, 31.4% cases of severe primary mitral regurgitation (MR), 7.5% cases of severe secondary MR, 43.7% cases of severe aortic stenosis, 27.5% cases of severe aortic regurgitation, and 7.2% cases of severe tricuspid regurgitation. The overall in-hospital mortality rate for patients with severe VHD was 5.4%, and for secondary severe MR and severe tricuspid regurgitation, the rates were 9.0% and 7.5%, respectively, indicating a poor prognosis. In-hospital mortality occurred in 73 of the 1,244 patients (5.9%) who received conservative treatment and in 18 of the 455 patients (4.0%) who received a surgical or transcatheter intervention, which was significantly lower in the intervention group (*P* = 0.037).

**Conclusions:**

This study provides important information about the current status of VHD diagnosis and treatment through a nationwide registry in Korea and helps to define future changes.

## Background

Valvular heart disease (VHD) accounts for a rapidly growing proportion of cardiovascular morbidity and mortality around the world [1]. Echocardiography has become an essential standard, not only for the diagnosis of VHD, but for determining treatment [2–5]. Although standard guidelines for diagnosing and treating VHD have been published in the United States and Europe, little is known about clinical situations in other countries.

The burden of VHD in Korea has changed significantly over the past 50 years due to the aging population, socioeconomic development, and advances in treatment [4, 5]. The burden of rheumatic valve disease has been dramatically reduced, and degenerative or secondary causes are now the leading cause of VHD [6–8]. Because the medical system in Korea is universally accessible, evaluating the effectiveness of echocardiography as an initial diagnostic tool for VHD is relatively straightforward, and VHD is often detected early.

The Korean Society of Echocardiography (KSE) implemented the Korean Valve Survey (KVS) registry as a major academic project, and the contemporary prevalence, etiology, and demographic profiles of VHD in Korea are reported in part 1 of the survey [8]. Part 2 focuses on patients with severe VHD who are eligible for consideration for surgical or percutaneous treatment. To examine diagnoses, we investigated the rates of key echocardiographic parameters and the implementation status of additional imaging for each case of severe VHD. In terms of treatment, we examined the rates and outcomes of surgery and transcatheter intervention in each case of severe VHD.

## Methods

### Study design and population

This nationwide, retrospective, multicenter, observational study of VHD was designed by the clinical practice guidelines committee of the KSE to investigate the position of VHD in Korea with the participation of 45 hospitals representing each region of the country. A list of participating sites and investigators is provided at the end.

Among patients aged 18 years and older who visited each participating hospital between September 1 and October 31, 2019, those with at least a moderate degree of VHD, as diagnosed by echocardiography, were included in this registry [8]. There was no limit on the number of enrolled patients at each center. Patients with severe native VHD were analyzed, and those previous surgical or transcatheter valve replacement or repair were excluded.

### Data collection

Data were collected using a password-protected, web-accessible electronic case report form (eCRF; https://kmcecrf.kr/vhd) created a priori by consensus agreement of the Committee of Clinical Practice Guidelines of the KSE. Each eCRF included demographics, clinical information, echocardiographic findings, additional investigations, and therapeutic decisions. In-hospital mortality and cause of death were also investigated. The collected data were coded and stored, and access to them was strictly controlled. The attending physicians completed the eCRFs with assistance from clinical research coordinators. Data in the eCRFs were audited by the two study investigators (JWS and JBP).

### Data analysis

A diagnosis of severe VHD was made based on echocardiograph results and an integrative approach following the VHD guidelines in effect at the time of enrollment. However, in severe mitral stenosis (MS), cutoffs of either 1.0 or 1.5 cm^2^ for the mitral valve area were according to each institution’s standards. We therefore reclassified patients with a mitral valve area of 1.5 cm^2^ or less as severe MS and included them in this study. A mitral valve area of 1.0 cm^2^ or less was separately classified as very severe MS. The reporting rates of echocardiographic parameters recommended in the guidelines for a diagnosis of severe VHD were investigated. The decision to perform further investigations, in the form of transesophageal echocardiography, stress echocardiography, cardiac computed tomography, coronary angiography, catheterization, or cardiac magnetic resonance, was made by the attending physicians at each center. Analysis was performed separately for each case of VHD. However, when severe dysfunction was observed in multiple valves in the same patient, the patient was included and analyzed each disease group.

### Statistical analysis

Categorical variables were presented as frequencies and percentages. A chi-square or Fisher exact test was performed to test for differences in categorical variables between groups. Continuous variables were presented as mean ± standard deviations. Student t-tests were performed to measure the differences in continuous variables between the two groups. *P*-values of < 0.05 were considered statistically significant. Data were analyzed in Stata ver. 16.0 (Stata Corp).

## Results

### Baseline characteristics

**Fig. 1:**
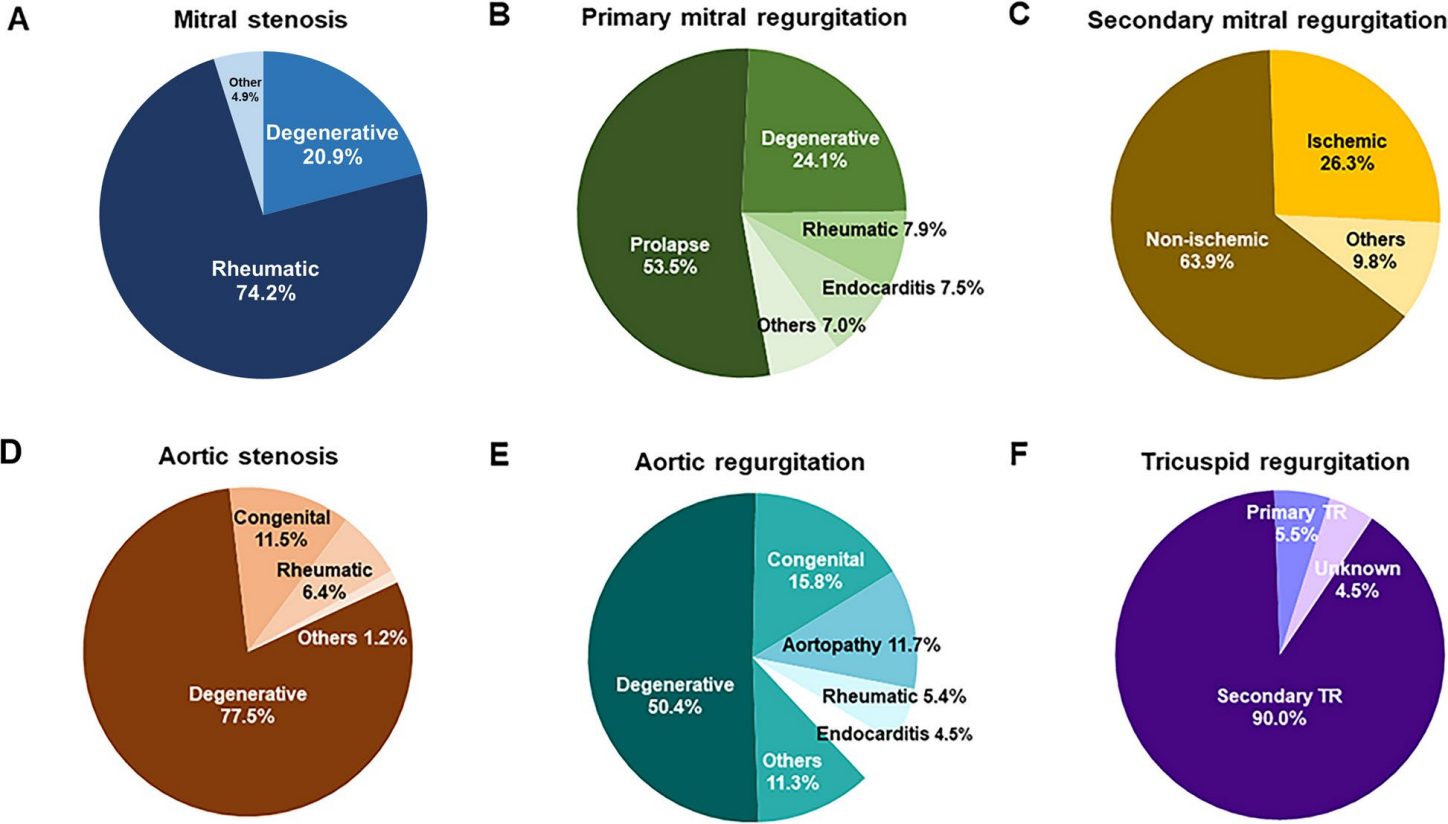
Etiology of each severe valvular heart disease in Korea. Pie charts showing the distribution of the etiology of (**A**) mitral stenosis, (**B**) primary mitral regurgitation, (**C**) secondary mitral regurgitation, (**D**) aortic stenosis, (**E**) aortic regurgitation, and (**F**) tricuspid regurgitation

Table 1 shows the clinical and echocardiographic characteristics of patients with severe VHD in Korea. Among 1,482 patients, 244 (16.5%) had severe MS, 229 (15.5%) had severe primary mitral regurgitation (MR), 133 (8.9%) had severe secondary MR, 551 (37.2%) had severe aortic stenosis (AS), 222 (15.0%) had severe aortic regurgitation (AR), and 320 (21.6%) had severe tricuspid regurgitation (TR). Similar to the demographic characteristics of each case of VHD of at least a moderate degree shown in part 1 [8], patients with severe MS, severe primary MR, and severe AR were younger than those with severe secondary MR, severe AS, and severe TR. Most cases of severe MS and TR occurred in female patients, and most cases of severe AR occurred in males. Figure 1 shows the significant causes of each case of severe VHD and their proportions. Analysis of accompanying comorbidity showed characteristics according to the demographics of each VHD case, and the rate of atrial fibrillation was relatively high in cases of severe MS and severe TR.

**Table 1:** Demographic, clinical, and echocardiographic characteristics of patients with severe valvular heart disease in Korea

Characteristic	Severe MS (*n* = 244)	Severe primary MR (*n* = 229)	Severe secondary MR (*n* = 133)	Severe AS (*n* = 551)	Severe AR (*n* = 222)	Severe TR (*n* = 320)
Clinical characteristic						
Age (yr)	65.8 ± 11.1	65.0 ± 15.7	71.6 ± 13.4	76.9 ± 10.2	65.8 ± 14.1	72.3 ± 12.9
Male sex	78 (32.0)	108 (47.2)	69 (51.9)	284 (51.5)	138 (62.7)	143 (44.7)
Systolic blood pressure (mmHg)	119.3 ± 18.4	122.8 ± 18.8	115.9 ± 20.1	126.7 ± 20.1	128.5 ± 18.1	118.4 ± 18.4
Diastolic blood pressure (mmHg)	70.5 ± 12.1	71.7 ± 13.2	68.4 ± 12.0	69.2 ± 12.6	65.2 ± 12.6	69.8 ± 12.8
Body mass index (kg/m^2^)	23.3 ± 3.5	23.5 ± 3.9	22.9 ± 3.4	23.6 ± 3.6	23.2 ± 3.7	23.4 ± 4.1
History of smoking	19 (8.0)	17 (8.7)	8 (6.6)	24 (4.4)	13 (7.5)	13 (4.7)
NYHA Functional Classification						
I	91 (38.6)	79 (64.6)	18 (13.5)	152 (28.4)	91 (41.4)	84 (26.2)
II	119 (50.4)	88 (38.6)	56 (42.1)	248 (46.3)	81 (36.8)	125 (39.1)
III	20 (8.5)	46 (20.2)	33 (24.8)	106 (19.8)	37 (16.8)	74 (23.1)
IV	6 (2.5)	15 (6.6)	26 (19.6)	30 (5.6)	11 (5.0)	37 (11.6)
Unknown	8 (3.2)	1 (0.1)	0 (0)	15 (2.8)	2 (0.1)	0 (0)
NYHA class ≥ II	145 (59.4)	149 (65.4)	115 (86.5)	384 (69.8)	129 (58.6)	236 (73.8)
Hypertension	90 (37.3)	107 (46.9)	72 (54.1)	361 (65.6)	135 (61.6)	182 (57.1)
Diabetes	43 (17.8)	41 (18.0)	42 (31.8)	161 (29.3)	26 (11.9)	69 (21.6)
Dyslipidemia	53 (22.1)	35 (15.4)	22 (16.7)	180 (32.9)	38 (17.4)	76 (23.8)
Atrial fibrillation	160 (66.4)	76 (33.2)	66 (49.6)	80 (14.5)	37 (16.8)	222 (69.4)
Chronic dialysis	7 (2.9)	11 (4.8)	12 (9.0)	19 (3.5)	7 (3.2)	34 (10.6)
Chronic pulmonary disease	14 (5.8)	21 (9.3)	16 (12.1)	51 (9.3)	13 (6.0)	50 (15.7)
Previous myocardial infarction	7 (2.9)	8 (3.5)	19 (14.3)	39 (7.1)	7 (3.2)	27 (8.4)
Hemoglobin (g/dL)	12.6 ± 2.2	12.3 ± 2.4	11.2 ± 2.1	11.8 ± 2.0	12.4 ± 2.3	11.2 ± 2.2
Creatinine (mg/dL)	1.1 ± 1.2	1.3 ± 1.7	1.7 ± 1.8	1.2 ± 1.1	1.2 ± 1.2	1.6 ± 1.6
Creatinine clearance (mL/min)	70.9 ± 23.4	70.2 ± 28.3	55.8 ± 33.0	66.7 ± 26.7	71.5 ± 27.1	55.2 ± 29.1
NT-proBNP (pg/mL)	3,790 ± 6,970	4,540 ± 9,668	10,763 ± 11,779	6,592 ± 10,766	7,714 ± 11,142	7,609 ± 13,123
Echocardiographic characteristic						
LVEDD (mm)	48.6 ± 6.5	56.4 ± 7.7	61.9 ± 9.9	49.1 ± 7.3	61.5 ± 7.9	49.7 ± 9.2
LVESD (mm)	32.2 ± 6.3	36.8 ± 8.2	49.0 ± 11.9	32.5 ± 8.5	43.1 ± 8.5	35.3 ± 10.1
LVEF (%)	58.7 ± 8.9	63.9 ± 10.1	42.2 ± 16.9	62.1 ± 13.4	59.3 ± 10.9	55.7 ± 14.6
IVS thickness (mm)	9.2 ± 1.8	9.5 ± 1.9	9.3 ± 1.8	11.4 ± 2.2	10.3 ± 2.0	9.1 ± 1.7
LV posterior wall thickness (mm)	9.1 ± 1.7	9.5 ± 1.6	9.0 ± 1.6	11.0 ± 2.0	10.3 ± 1.9	9.1 ± 1.6
LV mass index (g/m^2^)	97.7 ± 28.8	128.7 ± 38.1	146.5 ± 42.6	131.4 ± 40.9	163.5 ± 46.4	102.3 ± 36.0
TR Vmax (m/sec)	2.9 ± 0.5	3.0 ± 0.7	3.3 ± 0.6	2.8 ± 0.5	2.6 ± 0.6	3.0 ± 0.8
LA volume index (mL/m^2^)	89.3 ± 44.2	87.5 ± 56.7	91.7 ± 52.9	54.3 ± 26.0	56.1 ± 30.2	82.9 ± 53.4

Values are presented as mean ± standard deviation or number (%)

*MS* mitral stenosis, *MR* mitral regurgitation, *AS* aortic stenosis, *AR* aortic regurgitation, *TR* tricuspid regurgitation, *NYHA* New York Heart Association, *NT-proBNP* N-terminal pro B-type natriuretic peptide, *LVEDD* left ventricular end-diastolic dimension, *LVESD* left ventricular end-systolic dimension, *LVEF* left ventricular ejection fraction, *LV* left ventricle, *IVS* interventricular septum, *Vmax* maximal velocity, *LA* left atrium

### Reporting rate of echocardiographic parameters recommended for diagnosis

Table 2 presents the reporting rates of echocardiographic parameters in patients with severe VHD. In cases of severe MS, the mitral valve area using two-dimensional (2D) planimetry was reported for 95.5% of patients (233 of 244), and the mean diastolic pressure gradient was reported for 98.8% of patients (241 of 244). The mitral valve area, as calculated using pressure half-time, was also reported in 218 patients (89.3%). This confirmed that most of the parameters used for diagnosing severe MS were faithfully reported. In cases of severe AS, calculation of aortic valve area using the continuity equation, as recommended by current guidelines, was reported for 96.7% of cases. Aortic valve (AV) peak velocity and transvalvular pressure gradients were also reported.

**Table 2:** Reporting rate of echocardiographic parameters in patients with severe valvular heart disease

Parameter	Severe MS (*n* = 244)	Severe Primary MR (*n* = 229)	Severe Secondary MR (*n* = 133)	Severe AS (*n* = 551)	Severe AR (*n* = 222)	Severe TR (*n* = 320)
Valve area						
By 2D planimetry	233 (95.5)	-	-	201 (36.5)	-	-
By PHT	218 (89.3)	-	-	-	-	-
By continuity equation	-	-	-	533 (96.7)	-	-
Transvalvular pressure gradient	241 (98.8)	-	-	545 (98.9)	-	-
AV peak velocity	-	-	-	518 (94.0)	-	-
Velocity ratio	-	-	-	247 (44.8)	-	-
Central large jet > 50% of LA area	-	122 (53.3)	101 (75.9)	-	-	-
Pulmonary vein systolic flow reversal	-	68 (29.7)	41 (30.8)	-	-	-
Vena contracta width	-	26 (11.4)	20 (15.0)	-	101 (45.5)	89 (27.8)
PISA radius at Nyquist 30–40 cm/sec	-	190 (83.0)	123 (92.5)	-	37 (16.7)	144 (45.0)
EROA	-	149 (65.1)	79 (59.4)	-	39 (17.6)	24 (7.5)
Regurgitant volume	-	114 (49.8)	53 (39.8)	-	33 (14.9)	12 (3.8)
Regurgitant fraction	-	7 (3.1)	7 (5.3)	-	9 (4.1)	-
AR jet width to LVOT ratio (central jet)	-	-	-	-	104 (46.8)	-
AR jet CSA/LVOT CSA (central jet)	-	-	-	-	68 (30.6)	-
AR pressure half-time	-	-	-	-	110 (49.5)	-
DTA diastolic flow reversal	-	-	-	-	147 (66.2)	-
Central jet > 50% of RA	-	-	-	-	-	226 (70.6)
TR jet area	-	-	-	-	-	100 (31.3)
Systolic reversal of hepatic vein flow	-	-	-	-	-	187 (58.4)
Tricuspid inflow E velocity (m/sec)	-	-	-	-	-	21 (6.6)

*MS* mitral stenosis, *MR* mitral regurgitation, *AS* aortic stenosis, *AR* aortic regurgitation, *TR* tricuspid regurgitation, *D* dimensional, *PHT* pressure half-time, *PG* pressure gradient, *AV* aortic valve, *LA* left atrium, *PISA* proximal isovelocity surface area, *EROA* effective regurgitant orifice area, *LVOT* left ventricular outflow tract, *CSA* cross-sectional area, *DTA* descending thoracic aorta, *RA* right atrium

However, the reporting rate for each parameters used to diagnose the regurgitation of each valve was relatively low, which can be interpreted as characteristic of valvular regurgitation, making integrated decisions about multiple parameters necessary. For a diagnosis of severe MS, proximal isovelocity surface area (PISA) radius, effective regurgitant orifice area (EROA), and regurgitant volume appeared to be used most often, and for the diagnosis of severe AR, the reporting rate of descending thoracic aorta diastolic flow reversal was high at 66.2%, and vena contracta width, AR jet width to left ventricular outflow tract ratio, and AR pressure half-time were reported in approximately half of the cases. In severe TR, the central jet > 50% of the right atrium was reported in 66.8% of cases. Systolic reversal of the hepatic vein flow was the next most reported at 56.3%, and the vena contracta width was reported in 89 patients, which was relatively low, at 26.8%. Although the reporting rate of PISA radius in cases of severe TR was 43.4%, cases of calculating EROA and regurgitant volume were remarkably low at 7.2% and 3.6%, respectively.

### Additional imaging in diagnosis and treatment of severe VHD

**Fig. 2:**
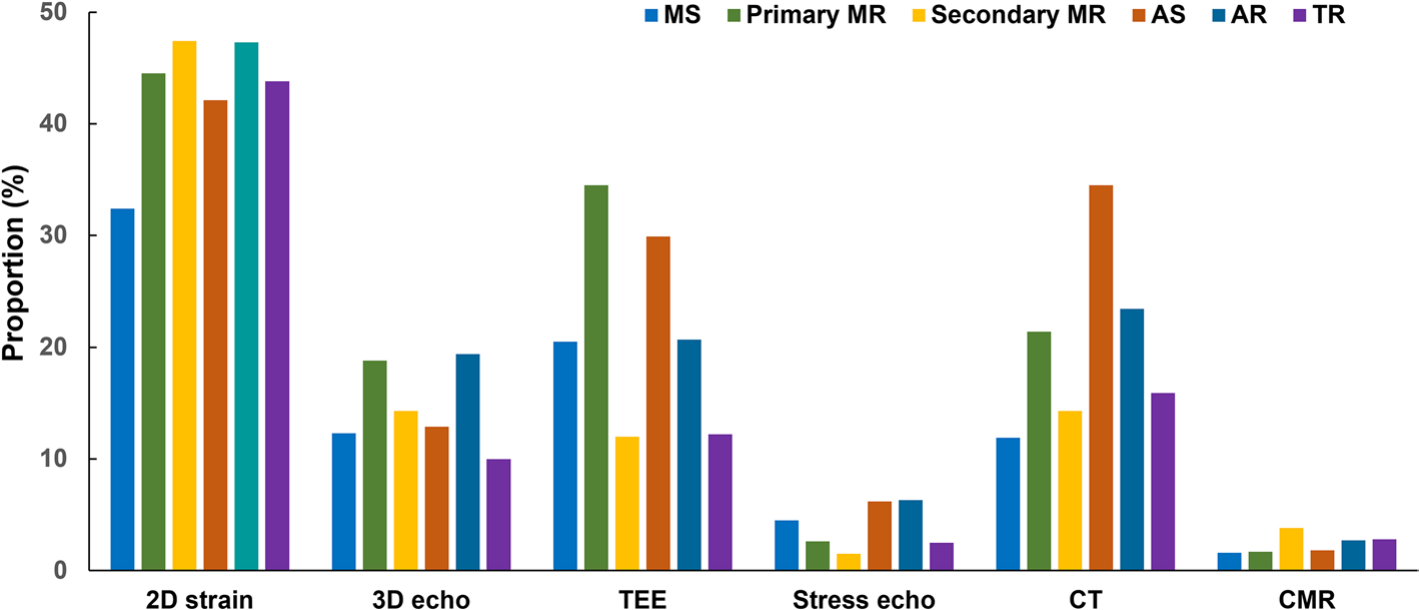
Approaches based on the multimodal imaging for each valvular heart disease. MS, mitral stenosis; MR, mitral regurgitation; AS, aortic stenosis; AR, aortic regurgitation; TR, tricuspid regurgitation; D, dimensional; TEE, transesophageal echocardiography; CT, computed tomography; CMR, cardiac magnetic resonance

With respect to imaging modalities performed in addition to conventional transthoracic echocardiography, speckle tracking echocardiography was the most frequently performed advanced echocardiographic technique (43.3%), followed by transesophageal echocardiography at 30.0% and cardiac computed tomography at 17.8%. Figure 2 shows the proportion of additional imaging methods used for each disease. Transesophageal echocardiography was frequently used in cases of severe primary MR and severe AS, and cardiac computed tomography had a high utilization rate (34.5%) for severe AS.

### Treatment and outcomes of severe VHD

Of the 1,482 patients with severe VHD, intervention in the relevant valve was performed in 455 patients (30.7%). Surgical or transcatheter intervention was performed in 47 patients (19.3%) with severe MS, 73 (31.9%) with severe primary MR, 10 (7.5%) with severe secondary MR, 241 (43.7%) with severe AS, 61 (27.5%) with severe AR, and 23 (7.2%) with severe TR. The overall in-hospital mortality rate for patients with severe VHD was 5.3% (79 of 1,482 patients). Looking at each valve, in-hospital mortality was high at 9.0% and 7.5% for cases of secondary severe MR and severe TR, respectively, reflecting the poor prognosis of these valve diseases. In-hospital mortality occurred in 73 of the 1,244 patients (5.9%) who received conservative treatment and in 18 of the 455 patients (4.0%) who received the surgical or transcatheter intervention, which was significantly lower in the intervention group (*P* = 0.037).

### Severe MS

Among 244 patients with severe MS, mitral valve replacement (MVR) was performed in 37 patients (15.2%) and percutaneous balloon mitral valvuloplasty (PMV) was performed in 10 patients (4.1%). In a comparison of treatment strategies, patients undergoing PMV were significantly younger than those in other groups (*P* = 0.005) (Table 3). The proportion of patients with New York Heart Association (NYHA) class II or greater symptoms and atrial fibrillation tended to be higher in the MVR and PMV groups compared with the conservative group, but no statistical significance was seen in differences among the three groups. Patients who underwent PMV tended to have a higher body mass index, lower creatinine levels, and higher creatinine clearance than did patients in other groups.

**Table 3:** Demographic, clinical, and echocardiographic characteristics in patients with severe MS

Characteristic	Severe MS				
	Total (*n* = 244)	Conservative (*n* = 197)	MVR (*n* = 37)	PMV (*n* = 10)	*P*-value^a^
Clinical characteristic					
Age (yr)	65.8 ± 11.1	66.6 ± 11.1	63.8 ± 9.8	55.4 ± 8.7	0.005^*^
Male sex	78 (32.0)	68 (34.5)	8 (21.6)	2 (20.0)	0.229
Body mass index (kg/m^2^)	23.3 ± 3.5	23.2 ± 3.3	23.7 ± 4.2	25.6 ± 3.0	0.055
NYHA class ≥ II	145 (59.4)	111 (56.3)	27 (73.0)	7 (70.0)	0.140
Hypertension	90 (36.9)	74 (37.6)	15 (40.5)	1 (10.0)	0.195
Diabetes	43 (17.6)	33 (16.8)	10 (27.0)	0 (0)	0.124
Dyslipidemia	53 (21.7)	44 (22.3)	7 (18.9)	2 (20.0)	0.900
Atrial fibrillation	160 (65.6)	126 (64.0)	27 (73.0)	7 (70.0)	0.670
Chronic dialysis	7 (2.9)	6 (3.1)	1 (2.7)	0 (0)	0.999
Chronic pulmonary disease	14 (5.8)	10 (5.2)	3 (8.1)	1 (10.0)	0.613
Previous MI	7 (2.9)	7 (3.6)	0 (0)	0 (0)	0.763
Hemoglobin (g/dL)	12.6 ± 2.2	12.5 ± 2.3	13.0 ± 2.0	13.5 ± 1.8	0.310
Creatinine (mg/dL)	1.1 ± 1.2	1.1 ± 1.1	1.3 ± 1.6	0.8 ± 0.1	0.024^*^
Creatinine clearance (mL/min)	70.9 ± 23.4	71.7 ± 24.8	63.8 ± 17.9	81.7 ± 12.3	0.032^*^
NT-proBNP (pg/mL)	3,790 ± 6,970	3,523 ± 6,273	6,777 ± 11,661	1,277 ± 1,379	0.468
Echocardiographic characteristic					
LVEDD (mm)	48.6 ± 6.5	49.0 ± 6.6	47.3 ± 6.8	46.4 ± 3.1	0.216
LVESD (mm)	32.2 ± 6.3	32.4 ± 6.5	31.2 ± 6.1	32.0 ± 4.3	0.692
LVEF (%)	58.7 ± 8.9	59.1 ± 8.7	57.7 ± 10.6	55.1 ± 5.6	0.121
TR Vmax (m/sec)	2.9 ± 0.5	2.9 ± 0.5	3.1 ± 0.6	3.2 ± 0.5	0.104
LA volume index (mL/m^2^)	89.3 ± 44.2	87.7 ± 41.3	101.4 ± 57.8	71.0 ± 29.1	0.414
MS etiology					
Rheumatic	181 (74.2)	140 (71.1)	33 (89.2)	8 (80.0)	0.485
Degenerative	51 (20.9)	45 (22.8)	4 (10.8)	2 (20.0)	
MVA by 2D planimetry (cm^2^)	1.16 ± 0.24	1.18 ± 0.24	1.02 ± 0.19	1.11 ± 0.28	< 0.001^*^
Very severe MS	62 (25.4)	41 (20.8)	16 (43.2)	5 (50.0)	0.003^*^
MVA by PHT (cm^2^)	1.23 ± 0.26	1.26 ± 0.26	1.09 ± 0.25	1.07 ± 0.19	< 0.001^*^
Mean PG (mmHg)	8.0 ± 3.7	7.6 ± 3.5	9.3 ± 3.3	12.6 ± 4.7	< 0.001^*^
Treatment strategy					
Mechanical MVR	25 (10.2)	0 (0)	25 (67.6)	0 (0)	-
Bioprosthetic MVR	12 (4.9)	0 (0)	12 (32.4)	0 (0)	-
PMV	10 (4.1)	0 (0)	0 (0)	10 (100)	-
In-hospital mortality	5 (2.0)	3 (1.6)	2 (5.4)	0 (0)	-
Cardiac	0 (0)	0 (0)	0 (0)	0 (0)	
Noncardiac	4 (1.6)	2 (1.0)	2 (5.4)	0 (0)	
Unknown	1 (0.4)	1 (0.5)	0 (0)	0 (0)	

Values are presented as mean ± standard deviation or number (%)

*MS* mitral stenosis, *MVR* mitral valve replacement, *PMV* percutaneous mitral valvuloplasty, *NYHA* New York Heart Association, *MI* myocardial infarction, *NT-proBNP* N-terminal pro B-type natriuretic peptide, *LVEDD* left ventricular end-diastolic dimension, *LVESD* left ventricular end-systolic dimension, *LVEF* left ventricular ejection fraction, *TR* tricuspid regurgitation, *Vmax* maximal velocity, *LA* left atrium, *MVA* mitral valve area, *D* dimensional, *PHT* pressure half-time, *PG* pressure gradient

^a^Kruskal-Wallis rank test, Fisher exact test

^*^P < 0.05

In terms of echocardiographic characteristics, the transvalvular mean diastolic pressure gradient was significantly higher in the PMV group (12.6 ± 4.7 mmHg, *P* < 0.001) than in the conservative group (7.6 ± 3.5 mmHg) and MVR group (9.3 ± 3.3 mmHg). The mitral valve area as measured by 2D planimetry was smaller in patients who underwent MVR. However, the proportion of cases of very severe MS was greater in patients who underwent PMV. This result can be explained by the fact that PMV is performed in younger patients with an anatomically suitable condition and high transvalvular pressure gradient or symptomatic patients.

Among patients who required MVR, 25 (67.6%) underwent mechanical valve surgery, and 12 (32.4%) underwent bioprosthetic valve replacement. In-hospital mortality occurred in five patients (2.0%), three patients (1.6%) in the conservative group, and two patients (5.4%) in the MVR group. It did not occur in the PMV group, and no cases of cardiac mortality were reported among patients with severe MS.

### Severe primary MR

Among 229 patients with severe primary MR, 73 (31.9%) underwent mitral valve intervention. Mitra l valve surgical repair was performed in 38 patients (16.6%), whereas surgical MVR was performed in 34 (14.8%). Primary MR patients who underwent mitral intervention were significantly younger, tended to be more symptomatic, and had fewer comorbidities compared with those who underwent conservative treatment (Table 4). In the intervention group, left ventricular end-diastolic dimension was significantly more significant than the conservative group, but there were no differences in left ventricular ejection fraction (LVEF), left atria volume index, and TR maximal velocity (Vmax). The most common etiology of severe primary MR was mitral valve prolapse (53.5%), and there was no significant difference between the two groups. Regarding MR quantification parameters, the PISA radius and PISA radius-driven EROA values were greater in the intervention group than in the conservative group. The in-hospital mortality rate was high in the conservative group, with nine patients (5.8%) in the conservative group and one patient (1.4%) in the intervention group, but not to a statistically significant level (*P* = 0.180).

**Table 4:** Demographic, clinical, and echocardiographic characteristics in patients with severe MR

Characteristic	Severe primary MR				Severe secondary MR			
	Total (*n* = 229)	Conservative (*n* = 156)	Intervention (*n* = 73)	*P*-value	Total (*n* = 133)	Conservative (*n* = 123)	Intervention (*n* = 10)	*P*-value^a^
Clinical characteristic								
Age (yr)	65.0 ± 15.7	67.7 ± 15.0	59.4 ± 15.9	< 0.001^*^	71.6 ± 13.4	71.7 ± 13.6	69.9 ± 10.1	0.324
Male sex	108 (47.2)	72 (46.1)	36 (49.3)	0.655	69 (51.9)	59 (48.0)	5 (50.0)	0.999
Body mass index (kg/m^2^)	23.5 ± 3.9	23.4 ± 4.0	23.8 ± 3.9	0.407	22.9 ± 3.4	22.7 ± 3.4	24.3 ± 3.5	0.127
NYHA class ≥ II	149 (65.1)	95 (60.9)	54 (74.0)	0.060	115 (86.5)	106 (86.2)	9 (90.0)	0.999
Hypertension	107 (46.7)	85 (54.5)	22 (30.1)	< 0.001^*^	72 (54.1)	65 (52.8)	7 (70.0)	0.342
Diabetes	41 (17.9)	32 (20.5)	9 (12.3)	0.127	42 (31.8)	41 (33.3)	1 (10.0)	0.164
Dyslipidemia	35 (15.2)	25 (16.0)	10 (13.7)	0.621	22 (16.7)	21 (17.1)	1 (10.0)	0.999
Atrial fibrillation	76 (33.2)	59 (37.8)	17 (23.3)	0.030^*^	66 (49.6)	61 (49.6)	5 (50.0)	0.999
Chronic dialysis	11 (4.8)	8 (5.2)	3 (4.1)	0.999	12 (9.0)	12 (9.8)	0 (0)	-
Chronic pulmonary disease	21 (9.3)	19 (12.3)	2 (2.8)	0.025^*^	16 (12.1)	14 (11.4)	2 (20.0)	0.344
Previous MI	8 (3.5)	4 (2.6)	4 (5.5)	0.276	19 (14.3)	17 (13.8)	2 (20.0)	0.635
Hemoglobin (g/dL)	12.3 ± 2.4	12.2 ± 2.2	12.6 ± 2.6	0.441	11.2 ± 2.1	11.1 ± 2.1	11.9 ± 2.2	0.260
Creatinine (mg/dL)	1.3 ± 1.7	1.4 ± 1.9	1.2 ± 1.2	0.636	1.7 ± 1.8	1.8 ± 1.8	1.0 ± 0.3	0.040^*^
Creatinine clearance (mL/min)	70.2 ± 28.3	66.0 ± 27.8	77.2 ± 28.1	0.013^*^	55.8 ± 33.0	54.0 ± 33.5	73.2 ± 21.7	0.080
NT-proBNP (pg/mL)	4,540 ± 9,668	5,666 ± 11,243	1,912 ± 2,873	0.018^*^	10,763 ± 11,780	10,975 ± 11,964	5,813 ± 4,275	0.868
Echocardiographic characteristic								
LVEDD (mm)	56.4 ± 7.7	55.4 ± 8.0	58.3 ± 6.8	0.004^*^	61.9 ± 9.9	62.1 ± 10.2	58.2 ± 5.7	0.225
LVESD (mm)	36.8 ± 8.2	36.4 ± 8.7	37.6 ± 7.0	0.062	49.0 ± 11.9	49.4 ± 12.0	43.2 ± 8.2	0.111
≥ 40	53 (23.2)	33 (21.3)	20 (27.4)	0.308	-	-	-	-
> 70	-	-	-	-	10 (7.5)	10 (8.1)	0 (0)	-
LVEF (%)	63.9 ± 10.1	63.5 ± 9.7	64.9 ± 11.4	0.174	42.2 ± 16.9	38.2 ± 15.1	49.5 ± 15.3	0.027^*^
≤ 60	84 (35.6)	61 (37.0)	23 (32.4)	0.600	119 (89.5)	113 (91.9)	6 (60.0)	0.011^*^
LV mass index (g/m^2^)	128.7 ± 38.1	128.8 ± 37.5	128.4 ± 39.5	0.929	146.5 ± 42.6	148.0 ± 42.4	127.8 ± 42.2	0.002^*^
TR Vmax (m/sec)	3.0 ± 0.7	2.9 ± 0.6	3.1 ± 0.7	0.147	3.3 ± 0.6	3.3 ± 0.6	3.3 ± 0.6	0.863
LA volume index (mL/m^2^)	87.5 ± 56.7	88.8 ± 61.5	84.5 ± 44.6	0.552	91.7 ± 52.9	93.3 ± 54.4	77.0 ± 36.2	0.383
MR etiology				0.170				0.007^*^
Degenerative	55 (24.0)	41 (6.3)	14 (19.4)		0 (0)	0 (0)	0 (0)	
Rheumatic	20 (8.7)	15 (9.6)	5 (6.9)		0 (0)	0 (0)	0 (0)	
Mitral valve prolapse	122 (53.2)	81 (51.9)	41 (56.9)		0 (0)	0 (0)	0 (0)	
Endocarditis	17 (7.4)	8 (5.1)	9 (12.5)		0 (0)	0 (0)	0 (0)	
Ischemic	0 (0)	0 (0)	0 (0)		35 (26.3)	32 (26.0)	3 (30.0)	
Nonischemic	0 (0)	0 (0)	0 (0)		85 (63.9)	82 (66.7)	3 (30.0)	
Other	15 (6.5)	12 (7.7)	3 (4.2)		13 (9.8)	9 (7.3)	4 (40.0)	
Central large jet > 50% of LA area	87 (71.3)	54 (67.5)	33 (78.6)	0.199	84 (83.2)	77 (82.8)	7 (87.5)	0.744
PV systolic flow reversal	36 (52.9)	25 (59.5)	11 (42.3)	0.167	23 (56.1)	19 (52.8)	4 (80.0)	0.070
Vena contracta width (cm)	0.69 ± 0.15	0.71 ± 0.16	0.66 ± 0.14	0.428	0.72 ± 0.11	0.73 ± 0.11	0.68 ± 0.11	0.393
PISA radius at Nyquist 30–40 cm/sec	1.05 ± 0.26	1.00 ± 0.23	1.15 ± 0.28	< 0.001^*^	0.91 ± 0.19	0.91 ± 0.19	1.01 ± 0.21	0.087
EROA (cm^2^)	0.54 ± 0.25	0.49 ± 0.20	0.66 ± 0.31	0.003^*^	0.43 ± 0.15	0.42 ± 0.15	0.50 ± 0.16	0.488
Regurgitant volume (mL/beat)	71.5 ± 24.3	68.7 ± 23.3	77.4 ± 25.6	0.085	64.1 ± 42.3	62.2 ± 42.2	87.4 ± 41.6	0.117
Regurgitant fraction (%)	56.5 ± 13.8	55.1 ± 16.6	60.1 ± 2.7	0.845	61.9 ± 0.0	59.1 ± 24.3	79.0 ± 0.0	0.574
Treatment strategy								
Mechanical MVR	17 (7.4)	0 (0)	17 (23.3)	-	3 (2.2)	0 (0)	3 (27.3)	-
Bioprosthetic MVR	17 (7.4)	0 (0)	17 (23.3)	-	1 (0.7)	0 (0)	1 (9.1)	-
MV surgical repair	38 (16.6)	0 (0)	38 (52.1)	-	5 (3.8)	0 (0)	5 (45.4)	-
Other	0 (0)	0 (0)	0 (0)	-	1 (0.7)	0 (0)	1 (9.1)	-
In-hospital mortality	10 (4.4)	9 (5.8)	1 (1.4)	0.180	12 (9.0)	11 (9.0)	1 (9.1)	0.999
Cardiac	3 (1.3)	3 (1.9)	0 (0)		8 (6.0)	8 (6.5)	0 (0)	
Noncardiac	6 (2.6)	6 (3.8)	0 (0)		4 (3.0)	3 (2.5)	1 (9.1)	
Unknown	1 (0.4)	0 (0)	1 (1.4)		0 (0)	0 (0)	0 (0)	

Values are presented as mean ± standard deviation or number (%)

*MR* mitral regurgitation, *NYHA* New York Heart Association, *MI* myocardial infarction, *NT-proBNP* N-terminal pro B-type natriuretic peptide, *LVEDD* left ventricular end-diastolic dimension, *LVESD* left ventricular end-systolic dimension, *LVEF* left ventricular ejection fraction, *LV* left ventricle, *TR* tricuspid regurgitation, *Vmax* maximal velocity, *LA* left atrium, *PV* pulmonary vein, *PISA* proximal isovelocity surface area, *EROA* effective regurgitant orifice area; MVR, mitral valve replacement; MV, mitral valve

^a^Wilcoxon rank sum test, Fisher exact test

^*^P < 0.05

### Severe secondary MR

Among 133 patients with severe secondary MR, 10 (7.5%) underwent mitral valve intervention. Mitral valve surgical repair was performed in five patients (50.0%), surgical MVR was performed in four patients (40.0%), and one patient (10.0%) underwent transcatheter intervention. Secondary MR patients undergoing mitral intervention did not differ in demographic and clinical profiles, except for a lower serum creatinine level (1.8 ± 1.8 mg/dL vs. 1.0 ± 0.3 mg/dL, *P* = 0.040) compared with patients without intervention (Table 4). LVEF was higher in the intervention group (38.2% ± 15.1% vs. 49.5% ± 15.3%, *P* = 0.027). The most common etiology of severe secondary MR was nonischemic origin (63.9%), but the proportion of patients with nonischemic origin in the intervention group was only 30%. In-hospital mortality during the observation period occurred in 12 patients (9.0%), which was 11 patients (9.0%) in the conservative group and one patient (9.1%) in the mitral intervention group (*P* = 0.999).

### Severe AS

Among 551 patients with severe AS, 241 (43.7%) underwent AV intervention. Surgical AV replacement (SAVR) was performed in 146 patients (26.5%), whereas transcatheter AV replacement (TAVR) was performed in 95 (17.2%). When comparing the three groups, patients with SAVR tended to be younger than those who received conservative care or TAVR (*P* = 0.073) (Table 5). The prevalence of patients with NYHA class II symptoms or more significant dyspnea was highest in the TAVR group, followed by the SAVR and conservative groups (*P* < 0.001).

**Table 5:** Demographic, clinical, and echocardiographic characteristics of patients with severe AS

Characteristic	Severe AS				
	Total (*n* = 551)	Conservative (*n* = 310)	SAVR (*n* = 146)	TAVR (*n* = 95)	*P*-value
Clinical characteristic					
Age (yr)	76.9 ± 10.2	79.2 ± 9.9	69.4 ± 9.2	80.9 ± 5.5	0.073
Male sex	284 (51.5)	166 (53.5)	66 (45.2)	52 (54.7)	0.723
Body mass index (kg/m^2^)	23.6 ± 3.6	23.3 ± 3.9	24.0 ± 3.1	23.8 ± 3.4	0.144
NYHA class ≥ II	384 (69.7)	188 (60.6)	112 (76.7)	84 (88.4)	< 0.001^*^
Hypertension	361 (65.5)	204 (66.0)	84 (57.5)	73 (76.8)	0.374
Diabetes	161 (29.2)	83 (26.8)	44 (30.1)	34 (35.8)	0.315
Dyslipidemia	180 (32.7)	95 (30.6)	47 (32.2)	38 (40.0)	0.198
Atrial fibrillation	80 (14.5)	47 (15.2)	20 (13.7)	13 (13.7)	0.650
Chronic dialysis	19 (3.5)	9 (2.9)	4 (2.7)	6 (6.3)	0.459
Chronic pulmonary disease	51 (9.3)	29 (9.4)	15 (10.3)	7 (7.4)	0.070
Previous MI	39 (7.1)	27 (8.7)	8 (5.5)	4 (4.2)	0.518
Hemoglobin (g/dL)	11.8 ± 2.0	11.6 ± 2.1	12.5 ± 1.9	11.3 ± 1.7	0.864
Creatinine (mg/dL)	1.2 ± 1.1	1.2 ± 1.1	1.2 ± 1.1	1.3 ± 1.3	0.729
Creatinine clearance (mL/min)	66.7 ± 26.7	65.9 ± 29.3	72.6 ± 22.6	61.8 ± 23.1	0.709
NT-proBNP (pg/mL)	6,592 ± 10,766	7,537 ± 11,281	4,404 ± 7,503	6,023 ± 8,687	0.185
Echocardiographic characteristic					
LVEDD (mm)	49.1 ± 7.3	48.1 ± 7.0	50.9 ± 7.5	48.8 ± 7.2	0.061
LVESD (mm)	32.5 ± 8.5	31.8 ± 8.3	33.9 ± 8.6	31.9 ± 8.6	0.396
LVEF (%)	58.3 ± 12.7	58.5 ± 12.7	57.8 ± 13.2	58.4 ± 12.3	0.804
≤ 50	113 (20.5)	59 (19.0)	35 (24.0)	19 (20.2)	0.474
LV mass index (g/m^2^)	131.4 ± 40.9	127.3 ± 40.5	137.2 ± 40.1	135.3 ± 42.1	0.008^*^
TR Vmax (m/sec)	2.8 ± 0.5	2.8 ± 0.5	2.7 ± 0.5	2.8 ± 0.5	0.513
LA volume index (mL/m^2^)	54.3 ± 26.0	53.5 ± 23.7	54.7 ± 30.7	56.5 ± 25.9	0.380
AS etiology					< 0.001^*^
Degenerative	427 (77.5)	245 (83.6)	88 (60.7)	94 (98.9)	
Rheumatic	35 (6.4)	23 (7.8)	12 (8.3)	0 (0)	
Congenital	64 (11.6)	21 (7.2)	42 (29.0)	1 (1.1)	
AV peak velocity (m/sec)	4.6 ± 0.8	4.4 ± 0.9	4.8 ± 0.7	4.7 ± 0.8	< 0.001^*^
AV mean PG (mmHg)	50.9 ± 19.1	48.5 ± 19.9	54.2 ± 16.5	54.8 ± 18.5	< 0.001^*^
AV area by 2D planimetry (cm^2^)	0.79 ± 0.23	0.80 ± 0.21	0.84 ± 0.30	0.72 ± 0.22	0.107
AV area by continuity equation (cm^2^)	0.73 ± 0.22	0.77 ± 0.23	0.70 ± 0.19	0.68 ± 0.20	< 0.001^*^
Velocity ratio	0.23 ± 0.09	0.2 ± 0.1	0.2 ± 0.1	0.2 ± 0.1	0.176
Treatment strategy					-
Mechanical AVR	52 (9.4)	0 (0)	52 (35.6)	0 (0)	
Bioprosthetic AVR	94 (17.1)	0 (0)	94 (64.4)	0 (0)	
TAVR	95 (17.2)	0 (0)	0 (0)	95 (100)	
In-hospital mortality	32 (5.8)	25 (8.1)	6 (4.1)	1 (1.1)	< 0.001^*^
Cardiac	9 (1.6)	9 (2.9)	0 (0)	0 (0)	
Noncardiac	21 (3.8)	16 (5.2)	4 (2.7)	1 (1.1)	
Unknown	2 (0.4)	0 (0)	2 (1.4)	0 (0)	

Values are presented as mean ± standard deviation or number (%)

*AS* aortic stenosis, *SAVR* surgical aortic valve replacement, *TAVR* transcutaneous aortic valve replacement, *NYHA* New York Heart Association, *MI* myocardial infarction, *NT-proBNP* N-terminal pro B-type natriuretic peptide, *LVEDD* left ventricular end-diastolic dimension, *LVESD* left ventricular end-systolic dimension, *LVEF* left ventricular ejection fraction, *LV* left ventricle, *TR* tricuspid regurgitation, *Vmax* maximal velocity, *LA* left atrium, *AV* aortic valve, *PG* pressure gradient, *D* dimensional, *AVR* aortic valve replacement

^*^P < 0.05

The most common etiology of severe AS was degenerative valve disease (77.5%), and the second most common etiology was congenital heart disease, including bicuspid AV (11.6%). Compared with the conservative group, the LV mass index, AV peak velocity, and mean pressure gradient were higher, and the AV area estimated by the continuity equation was smaller in the SAVR or TAVR group. In the SAVR group, 52 patients (35.6%) underwent SAVR with a mechanical valve, and 94 (64.4%) underwent bioprosthetic atrial valve replacement. In-hospital mortality in the conservative group (8.1%) was significantly higher than those in the SAVR (4.1%) and TAVR groups (1.1%, *P* < 0.001).

### Severe AR

Among 222 patients with severe AR, 61 (27.5%) underwent AV intervention. SAVR was performed in 55 patients, surgical AV repair was performed in three patients (4.9%), and TAVR was performed in three patients (4.9%) combined with significant AS. Demographics were similar between the conservative treatment and intervention groups, while more patients who underwent an AV intervention presented with NYHA functional class II or higher symptoms (Table 6). Patients in the intervention group had greater LV chamber size in both LV end-diastolic dimension (60.1 ± 7.4 vs. 65.4 ± 8.0, *P* < 0.001) and LV end-systolic volume (41.8 ± 7.9 vs. 46.7 ± 9.2, *P* < 0.001) compared with the conservative group. The intervention group had a statistically significant lower LVEF (55.8% ± 10.9% vs. 51.6% ± 12.5%, *P* = 0.014). As a result, the proportion of patients with LV end-systolic volume > 50 mm or LVEF ≤ 55% was 57.4% in the intervention group, greater than the 36.0% in the conservative group. LV mass index and TR Vmax were also higher in the intervention group compared with the conservative group. The most common etiology of severe AR was degenerative in both groups (55.9% vs. 36.1%). However, the intervention group had a relatively low rate of degenerative, rheumatic, or congenital etiologies, and a high rate of aorta pathology and endocarditis. Regarding AR measurement variables, AR pressure half-times tended to be shorter in the intervention group, and there were no differences in other variables. In-hospital mortality was not statistically different between groups but tended to be higher in the intervention group (1.9% vs. 8.2%, *P* = 0.063).

**Table 6:** Demographic, clinical, and echocardiographic characteristics of patients with severe AR

Characteristic	Severe AR			
	Total (*n* = 222)	Conservative (*n* = 161)	Intervention (*n* = 61)	*P*-value
Clinical characteristic				
Age (yr)	65.8 ± 14.1	65.8 ± 14.7	65.3 ± 12.9	0.812
Male sex	138 (62.7)	61 (37.9)	21 (34.4)	0.748
Body mass index (kg/m^2^)	23.2 ± 3.7	23.0 ± 3.7	23.8 ± 3.4	0.170
NYHA class ≥ 2	129 (58.1)	83 (51.5)	47 (77.0)	0.001^*^
Hypertension	135 (60.8)	99 (61.5)	37 (60.7)	0.815
Diabetes	26 (11.7)	18 (11.2)	8 (13.1)	0.768
Dyslipidemia	38 (17.1)	33 (20.5)	5 (8.2)	0.059
Atrial fibrillation	37 (16.7)	28 (17.4)	10 (16.4)	0.999
Chronic dialysis	7 (3.2)	4 (2.5)	3 (4.9)	0.450
Chronic pulmonary disease	13 (5.9)	9 (5.6)	4 (6.6)	0.661
Previous MI	7 (3.2)	5 (3.1)	2 (3.3)	0.681
Hemoglobin (g/dL)	12.4 ± 2.3	12.5 ± 2.3	12.3 ± 2.2	0.649
Creatinine (mg/dL)	1.2 ± 1.2	1.2 ± 1.2	1.2 ± 1.0	0.966
Creatinine clearance (mL/min)	71.5 ± 27.1	71.3 ± 28.9	73.1 ± 22.9	0.709
NT-proBNP (pg/mL)	7,714.0 ± 11,142.0	8,993.9 ± 12,357.4	4,361.4 ± 6,076.6	0.033^*^
Echocardiographic characteristic				
LVEDD (mm)	61.6 ± 7.9	60.1 ± 7.4	65.4 ± 8.0	< 0.001^*^
LVESD (mm)	43.2 ± 8.5	41.8 ± 7.9	46.7 ± 9.2	< 0.001^*^
> 50	40 (18.0)	21 (13.0)	19 (31.1)	0.003^*^
LVEF (%)	54.7 ± 11.5	55.8 ± 10.9	51.6 ± 12.5	0.014^*^
≤ 55	88 (39.6)	55 (34.2)	33 (54.1)	0.011^*^
LVESD > 50 mm or LVEF ≤ 55%	93 (41.9)	58 (36.0)	35 (57.4)	0.006^*^
LV mass index (g/m^2^)	163.5 ± 46.4	158.0 ± 47.0	178.2 ± 41.8	0.004^*^
TR Vmax (m/sec)	2.6 ± 0.6	2.5 ± 0.5	2.8 ± 0.7	0.008^*^
LA volume index (mL/m^2^)	56.1 ± 30.2	52.5 ± 25.8	67.1 ± 39.1	0.030^*^
AR etiology				< 0.001^*^
Degenerative	112 (50.4)	90 (55.9)	22 (36.1)	
Rheumatic	12 (5.4)	10 (6.2)	2 (3.3)	
Congenital	35 (15.8)	28 (17.4)	7 (11.5)	
Aorta pathology	26 (11.7)	14 (8.7)	12 (19.7)	
Endocarditis	10 (4.5)	3 (1.9)	7 (11.5)	
Other	25 (11.3)	14 (8.7)	11 (18.0)	
AR jet width to LVOT ratio (central jet)				0.827
Mild (< 25)	2 (1.5)	1 (1.1)	1 (2.4)	
Moderate (25–64)	47 (34.6)	33 (34.7)	14 (34.1)	
Severe (≥ 65)	87 (64.0)	61 (64.2)	26 (63.4)	
AR jet CSA/LVOT CSA (central jet)				0.502
Mild (5–20)	6 (6.6)	5 (7.2)	1 (4.5)	
Moderate (21–59)	24 (26.4)	20 (29.0)	4 (18.2)	
Severe (≥ 60)	61 (67.0)	44 (63.8)	17 (77.3)	
AR PHT (msec)	366.9 ± 132.2	380.6 ± 122.9	324.8 ± 152.2	0.056
AR vena contracta width (cm)	0.64 ± 0.16	0.64 ± 0.15	0.65 ± 0.19	0.673
Regurgitant volume (mL/beat)	65.8 ± 27.2	66.2 ± 30.0	64.7 ± 17.5	0.897
Regurgitant fraction (%)	47.3 ± 12.0	44.7 ± 12.2	56.5 ± 6.4	0.244
EROA (cm^2^)	0.36 ± 0.15	0.35 ± 0.16	0.41 ± 0.14	0.393
DTA diastolic flow reversal	140 (95.2)	95 (94.1)	45 (97.8)	0.564
Treatment strategy				-
Mechanical AVR	27 (12.2)	0 (0)	27 (44.3)	
Bioprosthetic AVR	28 (12.6)	0 (0)	28 (45.9)	
AV surgical repair	3 (1.3)	0 (0)	3 (4.9)	
TAVR	3 (1.3)^a^	0 (0)	3 (4.9)	
In-hospital mortality	8 (3.6)	3 (1.9)	5 (8.2)	0.063
Cardiac	2 (0.9)	2 (1.2)	0 (0)	
Noncardiac	3 (1.4)	1 (0.6)	2 (3.2)	
Unknown	3 (1.4)	0 (0)	3 (4.9)	

Values are presented as mean ± standard deviation or number (%)

*AR* aortic regurgitation, *NYHA* New York Heart Association, *MI* myocardial infarction, *NT-proBNP* N-terminal pro B-type natriuretic peptide, *LVEDD* left ventricular end-diastolic dimension, *LVESD* left ventricular end-systolic dimension, *LVEF* left ventricular ejection fraction, *LV* left ventricle, *TR* tricuspid regurgitation, *Vmax* maximal velocity, *LA* left atrium, *LVOT* left ventricular outflow tract, *CSA* cross-sectional area, *PHT* pressure half-time, *EROA* effective regurgitant orifice area, *DTA* descending thoracic aorta, *AVR* aortic valve replacement, *TAVR* transcutaneous aortic valve replacement

^a^AR cases combined with aortic stenosis underwent TAVR

^*^P < 0.05

### Severe TR

Among 320 patients with severe TR, 23 (7.2%) underwent tricuspid valve (TV) intervention. Surgical TV replacement was performed in six patients, and 17 underwent tricuspid annuloplasty or valvuloplasty. Patients who underwent TV surgery were significantly younger and had fewer comorbidities with preserved renal function compared with patients who received conservative care (Table 7). The TR vena contracta width in patients who underwent TV surgery was significantly larger than in the conservative group (0.98 ± 0.22 cm vs. 0.82 ± 0.29 cm, *P* = 0.036). Additionally, the intervention group exhibited a higher incidence of hepatic vein systolic reversal and TR jets > 50% of the right atrium area. In-hospital mortality occurred in 22 patients (7.4%) in the conservative group and two patients (8.7%) in the intervention group. This result was not statistically significant (*P* = 0.827).

**Table 7:** Demographic, clinical, and echocardiographic characteristics of patients with severe TR

Characteristic	Severe TR			
	Total (*n* = 320)	Conservative (*n* = 297)	Intervention (*n* = 23)	*P*-value^a^
Clinical characteristic				
Age (yr)	72.3 ± 12.9	72.9 ± 13.0	65.1 ± 7.5	< 0.001^*^
Male sex	143 (44.7)	137 (46.1)	6 (26.1)	0.059
Body mass index (kg/m^2^)	23.4 ± 4.1	23.4 ± 4.2	23.9 ± 3.5	0.310
NYHA class ≥ II	235 (73.4)	217 (73.1)	18 (78.3)	0.556
Hypertension	182 (56.9)	176 (59.3)	6 (26.1)	0.002^*^
Diabetes	69 (21.6)	63 (21.2)	6 (26.1)	0.627
Dyslipidemia	75 (23.4)	66 (22.2)	9 (39.1)	0.069
Atrial fibrillation	222 (69.4)	205 (69.0)	17 (73.9)	0.642
Chronic dialysis	33 (10.3)	32 (10.8)	1 (4.3)	0.329
Chronic pulmonary disease	50 (15.6)	47 (15.8)	3 (13.0)	0.822
Previous MI	27 (8.4)	26 (8.8)	1 (4.3)	0.464
Hemoglobin (g/dL)	11.2 ± 2.2	11.1 ± 2.2	11.8 ± 2.2	0.160
Creatinine (mg/dL)	1.6 ± 1.6	1.6 ± 1.6	1.0 ± 0.3	0.084
Creatinine clearance (mL/min)	55.2 ± 29.1	54.5 ± 29.7	66.0 ± 17.3	0.038^*^
NT-proBNP (pg/mL)	7,609 ± 13,123	7,280.5 ± 12,165.4	5,422.5 ± 9,996.4	0.494
Echocardiographic characteristic				
LVEDD (mm)	49.6 ± 9.2	49.7 ± 9.3	48.7 ± 7.9	0.633
LVESD (mm)	35.2 ± 10.1	35.3 ± 10.2	34.3 ± 8.3	0.806
LVEF (%)	53.1 ± 14.5	52.9 ± 14.6	55.8 ± 13.0	0.310
LV mass index (g/m^2^)	102.1 ± 36.0	102.8 ± 36.4	93.7 ± 29.4	0.210
TR Vmax (m/sec)	3.0 ± 0.8	3.1 ± 0.8	2.8 ± 0.5	0.207
TAPSE (mm)	1.7 ± 0.5	1.6 ± 0.5	1.8 ± 0.6	0.654
TV S’ (cm/sec)	10.3 ± 3.9	10.2 ± 3.9	10.7 ± 2.9	0.552
LA volume index (mL/m^2^)	82.9 ± 53.4	81.3 ± 50.8	112.7 ± 86.7	0.369
TR etiology				
Functional	287 (90.0)	268 (90.2)	19 (82.6)	0.298
Primary	18 (5.6)	16 (5.4)	2 (8.7)	
Central jet > 50% of RA area	184 (57.5)	167 (56.2)	17 (73.9)	0.048^*^
PISA radius at Nyquist 30–40 cm/sec (cm)	0.93 ± 0.22	0.91 ± 0.21	1.03 ± 0.29	0.100
Jet area (cm^2^)	20.6 ± 8.0	20.9 ± 8.2	18.4 ± 5.9	0.385
Vena contracta width (cm)	0.83 ± 0.28	0.82 ± 0.29	0.98 ± 0.22	0.036^*^
Systolic reversal of hepatic vein flow	150 (46.9)	135 (45.5)	15 (65.2)	0.045^*^
Tricuspid inflow E velocity (m/sec)	1.1 ± 0.9	1.2 ± 1.0	0.9	-
EROA (cm^2^)	0.51 ± 0.19	0.5 ± 0.2	0.5 ± 0.0	0.673
Regurgitant volume (2D PISA) (mL/beat)	58.7 ± 26.5	59.1 ± 27.7	54.1	-
Treatment strategy				-
TV replacement	6 (1.9)	0 (0)	6 (26.1)	
Tricuspid annuloplasty	7 (2.2)	0 (0)	7 (30.4)	
Tricuspid valvuloplasty	10 (3.1)	0 (0)	10 (43.5)	
In-hospital mortality	24 (7.5)	22 (7.4)	2 (8.7)	0.827
Cardiac	5 (1.6)	5 (1.6)	0 (0)	
Noncardiac	19 (5.9)	17 (5.7)	2 (7.7)	

Values are presented as mean ± standard deviation or number (%)

*TR* tricuspid regurgitation, *NYHA* New York Heart Association, *MI* myocardial infarction, *NT-proBNP* N-terminal pro B-type natriuretic peptide, *LVEDD* left ventricular end-diastolic dimension, *LVESD* left ventricular end-systolic dimension, *LVEF* left ventricular ejection fraction, *LV* left ventricle, *Vmax* maximal velocity, *TAPSE* tricuspid annular plane systolic excursion, *TV* tricuspid valve, *RA* right atrium, *PISA* proximal isovelocity surface area, *EROA* effective regurgitant orifice area, *D* dimensional

^**a**^Wilcoxon rank sum test, Fisher exact test

^*^P < 0.05

## Discussion

The principal findings of this study are as follows: (1) in stenotic valve diseases such as severe MS and severe AS, the most accurate diagnoses were based on key parameters, but in regurgitant valve diseases such as severe MR, AR, and TR, the reporting rate of quantitative parameters was not sufficient, as expected; (2) surgical or transcatheter intervention was performed in 19.3% of cases of severe MS, 31.4% of cases of severe primary MR, 7.5% of cases of severe secondary MR, 43.7% of cases of severe AS, 27.5% of cases of severe AR, and 7.2% of cases of severe TR; and (3) the overall in-hospital mortality rate for patients with severe VHD was 5.4%. In-hospital mortality occurred in 73 of the 1,244 patients (5.9%) who received conservative treatment and 18 of the 455 patients (4.0%) who received surgical or transcatheter intervention, and it was significantly lower in the intervention group. This study is provides valuable statistical information on contemporary diagnosis, treatment, and in-hospital outcomes for severe VHD in Korea.

### Epidemiology and characteristics of severe VHD in Korea

The clinical characteristics of severe VHD in Korea did not differ significantly from those of significant VHD, as shown in part 1 [8]. Patients diagnosed with severe AS were older and had more comorbidities, such as hypertension and diabetes, compared with other cases of severe VHD patients. Patients with severe secondary MR often had symptoms of NYHA class II or higher, the highest levels of N-terminal prohormone of brain natriuretic peptide, and relatively high creatinine levels. In contrast, patients diagnosed with severe MS were younger, nearly 70% were female, and most had good systemic conditions with fewer underlying diseases.

Adverse cardiac remodeling is the primary determinant of prognosis in patients with VHD [9]. These myocardial and cardiac chamber changes are caused by volume/pressure factors and concomitant disease affected by the specific form of VHD [9–15]. In our registry, severe secondary MR, and severe AR presented with an enlarged left ventricle dimension. Decreases in ejection fraction were more pronounced in cases of severe secondary MR. Patients with severe AS showed increased thickness of the left ventricle wall, and atrial volume was greater in severe mitral valve disease and severe TR. Except for TR, TR Vmax was highest in cases of severe AR, followed by severe secondary MR.

The etiology of each severe VHD is no different than that described in part 1, which reported the etiology of significant VHD. In cases of MS, practical survey results showed that many institutions adhere to a definition of severe MS as being no larger than 1.0 cm^2^. The definition was therefore revised to 1.5 cm^2^ or less and applied uniformly, resulting in an increase in the number of severe patients within the registry [5]. Still, in each case of severe VHD, the main etiology of MS was rheumatic, that of primary MR was mitral valve prolapse, secondary MR was nonischemic cause, AS and AR were degenerative, and TR was functional. This main etiology is not expected to change in the near future. However, because the degenerative portion is likely to increase in all types of severe VHD, it can serve as a point of comparison for future changes in the epidemiology of VHD in Korea. As the number of newly occurring cases of rheumatic MS rapidly decreases, interest in degenerative MS related to mitral annular calcification and risk stratification is growing [5, 16–18].

### Diagnostic approaches for severe VHD in Korea

Echocardiography is an essential test for diagnosing VHD and for determining the prognosis and timing of intervention in patients with severe VHD because it evaluates the etiology, severity, cardiac remodeling, and hemodynamic consequences. The increased use of multimodality imaging has resulted in a significant improvement in our understanding of the complicated aspects of VHD in recent years [13, 19–21]. Although 2D echocardiography remains the most popular imaging modality, evaluation of patients with VHD requires multimodality imaging for in-depth investigation of the underlying mechanism of valve dysfunction, precise quantification of disease severity, and consideration of any extravalvular issues. Advances in both surgical and transcatheter procedures have resulted in an increased demand for precise multimodality imaging tools to aid in patient and procedure selection [22, 23]. However, no statistical data on how much it is actually used in nationwide practice is available. This study did not target patients for whom specific treatment was planned, and the results should be interpreted with the understanding that additional imaging rates were investigated in patients diagnosed with severe VHD at 45 hospitals nationwide. Transesophageal echocardiography was performed as additional imaging in 30% of cases, coronary angiography in 18.9%, and cardiac computed tomography in 17.8%, which is not considered to be a low rate. Speckle tracking echocardiography is applied in 43.3% of cases. This reflects a great need for speckle tracking echocardiography to be used clinically, as it is easy to perform alongside conventional echocardiography and has several proven prognostic implications for patients with severe VHD [15, 24–27].

In addition, current guidelines emphasize an integrated diagnostic approach that comprehensively applies various parameters related to each case of VHD [2, 3, 28, 29]. However, it is challenging to measure and report multiple parameters. The reporting rate data for each major echocardiographic parameter shown through the KVS is significant because it reflects the current practice of echocardiographic assessment in VHD. Because the KVS systemically collected and analyzed echocardiographic data from 45 major university hospitals or hospitals over a specific period, and all participating institutions have echocardiologists certified in echocardiography by the KSE, any interpretation should assume the data are reliable.

### Treatment approaches for severe VHD in Korea

The main treatment strategies for patients with severe VHD are conservative or interventional treatment, which includes surgical treatment and transcatheter intervention [2–5]. As both surgical and transcatheter intervention showed advances in choices of treatment strategy, favorable data for early intervention for severe VHD has recently accumulated [30–32], and the role of imaging for successful intervention is being emphasized [22, 23]. Representative transcatheter interventions for severe VHD currently available in Korea include PMV, mitral transcatheter edge-to-edge repair (TEER), and transcatheter AV implantation. In addition, it is expected that various interventions, including tricuspid TEER for severe TR, transcatheter MVR for severe MS, or mixed mitral valve disease, will be possible soon. In addition, the use of mechanical valves is expected to decrease, even in surgical valve replacement, as valve-in-valve procedures become possible via a transcatheter approach in cases of structural degeneration of bioprosthetic valves. In other words, surgical MVR or PMV is currently applied as an intervention method in severe MS, but transcatheter MVR is expected to be used more often in the future. In severe TR, the intervention group underwent all surgical interventions, but the transcatheter approach will also be applied to the disease. In this study, the overall in-hospital mortality of the intervention group was lower than that of conservative treatment, which can be interpreted in various ways. In most comparative trials of intervention and conservative treatment for severe VHD, the early outcome (i.e., 30-day mortality) in the intervention group tends to be worse, but the long-term prognosis improved. Because this study is retrospective in design and may be subject to various confounding factors associated with the intervention, a simple comparison cannot be made. However, the significant improvement in in-hospital mortality in the intervention group, particularly in patients with severe AS, can be accepted as meaningful.

### Limitations

This study had several limitations. First, because it was a cross-sectional study, we were unable to determine any temporal patterns in prevalence and incidence. Second, despite our best efforts to identify the causes of VHD, it is difficult to assign a definitive etiology to any VHD cases due to the limited number of patients for whom surgical specimens were available. Third, referral bias may have affected our findings; because most participant institutions were universities or referral hospitals, the data may include more severe cases that required hospitalization or intervention. Fourth, although information was collected nationwide from 45 centers, differences at each institution may exist, and caution is needed when interpreting the results. Fifth, our data lacked specific information regarding the indications and symptoms of VHD patients, and other parameters were missing for some patients. Last, the clinical outcome in this study was in-hospital mortality, which provides no information on other meaningful outcomes, such as long-term survival or quality of life.

## Conclusions

This Korean national hospital-based registry study supplied up-to-date statistics on clinical and echocardiographic characteristics of severe VHD. This study provides important information on the current status of diagnosis and treatment of severe VHD in Korea and helps to define future changes.

## Data Availability

The datasets used and/or analyzed during the current study are available from the corresponding author on reasonable request.

## Abbreviations

AR: Aortic regurgitation
AS: Aortic stenosis
AV: Aortic valve
D: Dimensional
eCRF: Electronic case report form
EROA: Effective regurgitant orifice area
KSE: Korean Society of Echocardiography
KVS: Korean Valve Survey
LA: Left atria
LV: Left ventricular
LVEF: Left ventricular ejection fraction
MR: Mitral regurgitation
MS: Mitral stenosis
MVR: Mitral valve replacement
NYHA: New York Heart Association
PISA: Proximal isovelocity surface area
PMV: Percutaneous balloon mitral valvuloplasty
SAVR: Surgical aortic valve replacement
TAVR: Transcatheter aortic valve replacement
TEER: Transcatheter edge-to-edge repair
TR: Tricuspid regurgitation
TV: Tricuspid valve
VHD: Valvular heart disease
Vmax: Maximal velocity
